# Supplementary material for: Towards automated recipe genre classification using semi-supervised learning
Source: PLoS One. 2025 Jan 28;20(1):e0317697. doi: 10.1371/journal.pone.0317697 (PMC11774350; doi:10.1371/journal.pone.0317697)
Supplement: Attachment — Submitted filename: Response To Reviewers PONE-D-24-34319.pdf [file pone.0317697.s001.pdf]

Response to reviewers' comments of the Journal paper, PONE-D-24-34319		
**Reviewer # 1**		
**Reviewer's Comment to be addressed**	**Response/Action Taken**	**Manuscript reference**
1. The authors need to be more explicit about what is previously published and what is novel, especially with regards to the 3A2M dataset. In particular, these two quotations seem to contradict each other and require more clarity:	We sincerely thank the reviewer for their insightful comment. We appreciate the opportunity to clarify the distinct contributions of our dataset:<br><br>We have rewritten those paragraphs so that they are clear and concise.	
Page 5 Paragraph 3:<br>"The 3A2M dataset [9] is based on the RecipeNLG dataset and incorporates all of its data and features. The data attributes include title, directions, NER and genre as labels"	The 3A2M dataset [9] has been derived from the RecipeNLG dataset. While the RecipeNLG dataset contains recipe titles, directions, and Named Entity Recognition (NER) attributes without classification labels. So we have extended the ReceipeNLG dataset by adding genre as an important category.	Section 2.1<br>Page 4<br>Line 140-143
Page 7 Paragraph 2:<br>"The 3A2M dataset [9] contains a vast collection of 2,231,142 culinary recipes, making it the largest publicly available dataset of its kind. One lim-itation of the dataset is that it lacks specific genre categorization for the recipes."	The 3A2M dataset [9] is derived from RecipeNLG, and contains a vast collection of 2,231,142 culinary recipes, making it the largest publicly available dataset of its kind. One of the limitations of RecipeNLG was the absence of specific genre categorization for the recipes, that is what the 3A2M dataset contains through the human annotation and active learning techniques.	Section 3.1<br>Page 6<br>Line 217-221
2. What is the problem with the 3A2M genre labels?<br><br>Also, where do the 3A2M+ dataset genre labels come from? Are they inherited from 3A2M? How accurate were they originally in 3A2M? If your models are more consistent with the human labelled dataset, did you update the 3A2M+ genre labels?	Thank you for your valuable comment. We have specifically addressed this issue in Section 3.3, Dataset Properties, and Section 3.4, 3A2M and 3A2M+ Dataset Comparison.<br><br>The 3A2M+ dataset inherits the genre labels directly from the 3A2M dataset. As we have already published the 3A2M dataset [9] with those 9 genres, there is no problem with the genre. The accuracy of 3A2M genre labels is high because they are annotated by experts. Though a subset (300,000) of recipes were annotated by experts and the rest 1.9 million recipes were annotated by the active learning (and Query-by-Committee) approach, this approach was found to be a reliable annotation process in the literature [18] for a vast amount of	Section 3.3<br>Page 8<br>Line 294-300<br><br>Section 3.4<br>Page 9<br>Line 306-314

	dataset. The only difference between 3A2M and 3A2M+ is that the former does not contain specific details (e.g. cooking temperatures, cooking times, ingredient variations, quantity, etc.) about entities, and the latter, extends the NER feature by adding those specific entity details as shown in Table 5. We believe these are important for the accurate classification of recipes based on genre.	
3. Furthermore, the original 3A2M dataset had human labelled data and machine labelled data. For all experiments, the authors use machine labelled data to train their network and test against human labelled data. Is there a limitation by the fact machine learning models are trained on data generated by previous machine learning models, rather than just the human labelled dataset?	Thank you once again for your valuable feedback.<br><br>We acknowledge the fact that our machine-learning models are trained on machine-labeled data generated by previous machine-learning methods rather than solely on human-labeled data.<br><br>The 3A2M dataset was developed from the RecipeNLG dataset, which did not originally include genre categorization. To address this, three annotators, with guidance from a domain expert, labeled 300,000 recipes by genre. To construct the 3A2M dataset, we annotated 1.9 million recipes through an active learning approach with a Query-by-Committee (QBC) strategy, which may introduce minor inconsistencies compared to fully human-labeled data. We believe human-labeled subsets are exclusive high-quality data by which we can objectively evaluate the model performance and prevent any risk of data leakage or training bias. This is how we maintain the integrity of the testing phase and validate the model's performance in a real-world scenario, independent of training data.	Section 3.4<br>Page 9<br>Line 306-314
4. Since the extended NER list includes the original NER list, the authors should state how the original NER list was created. This will allow the method to be extended to other databases or other genre labels.	Thank you for this insightful comment.<br><br>We would like to clarify that the original NER list was not created by us; rather, it was adopted directly from the RecipeNLG dataset for use in 3A2M. In the extended 3A2M+ dataset, however, we expanded this list to incorporate additional named entities, enhancing the dataset's utility for more specific categorization tasks.	Section 3.3<br>Page 8<br>Line 294-300
5. Page 18, Figure 3 and 4: The labels in the legend are too long. You can put "Precision-recall curves" in the figure caption and expand on it for clarity and simply label each curve by number. If I understand correctly, the 9	Thank you for your valuable feedback. We appreciate your observation regarding Figure 3 and Figure 4.<br><br>We have revised these figures by shortening the labels in the legend to improve readability, as suggested. Additionally, we have expanded the	Section 6.1<br>Page 16

classes are the recipe genres, so it may be worth explicitly writing those in the legend. Furthermore, these figures are not discussed in depth in the text. What effect do they have on your results?	figure captions for clarity and explicitly noted the nine recipe genres in the legend.	
6. Page 19, Figure 5 and 6: Axis labels are missing. I assume Accuracy against Epoch? If that is the case, I don't understand why the validation accuracy is so high initially before training has begun, and plateaus while training. I also don't understand why the validation loss gets worse while training either. Is this because you're training on machine labeled data but testing on human labeled data? It's also unclear exactly which model this is for. Furthermore, you said there were only 10 epochs of training, but Figure 5 goes to 20 on the x-axis. These figures need clarification in the main text or caption.	Thank you very much for identifying this issue. We apologise for the oversight; the figures shown were mistakenly added from our initial experiments, as they shared the same file names across different folders. We have now updated the figures to reflect the correct experimental results and clarified these points in the main text and captions.	Section 6.1<br>Page 16-17
7. Page 25 Limitations, second point:<br>"Domain experts resolved tie situations. If the study was to redo the Extended<br>NER process for the entire 1900K dataset, it would make the dataset more<br>Robust."<br><br>Does this mean the Extended NER process was not performed for the full 3A2M dataset? Or is this about not generating genre labels based on Extended NER? The sentence needs to be clarified or expanded.	Thank you for your thoughtful question.<br><br>To clarify, the Extended NER process was indeed applied to the entire 3A2M dataset. During the initial creation, we labeled 1.9 million recipes using a machine learning process based on the original NER only. If we were to redo this process with the Extended NER (which includes additional entities such as cooking methods, times, temperatures, ingredients, quantities, and tools), it would make the 3A2M+ dataset even more robust. The original sentence was intended to highlight this potential enhancement.<br><br>We have rephrased the text accordingly.	Section 7<br>Page 22<br>Line 698-704

8. The motivation and conclusion for genres in recipes says it can be used for identifying diet restrictions or the origin of dishes, however the specific genres used don't seem designed for these and there's no connection to these uses.<br>I understand this is a limitation of the original 3A2M dataset. It could be possible to connect the results to the current conclusion by, for example, commenting on the recall/ROC of the vegetarian class and the implications for people on vegetarian diets. Otherwise, proposing a way to extend the dataset to different genre labels, which would make the extended NER list look more useful. Otherwise, the conclusion should be written closer to the specific uses of the current genre labels, e.g. recipe prediction, menu selection and explicitly mention the limitations.	Thank you for your valuable suggestion. We have updated the conclusion to better reflect the specific contributions of the current genre labels, including applications such as recipe prediction and menu selection.	Section 8<br>Page 22-23<br>Line 709-713
9. Some typographical errors I noticed:<br><br>Page 23 Section 6.5<br>"the Directionsfeature."<br>missing space	Thank you for pointing this out. We have corrected the missing space in "Directionsfeature."	Section 6.5<br>Page 21<br>Line 636
10. Page 24<br>"In the Experiment II, by analyzing the title with NER, and title with Extended NER using the DistilBERT model, this experiment was obtained 98% and 99y% accuracy in training respectively" 99% has extra y	Thank you for noting this typographical error. We have corrected "99y%" to "99%" in the text.	Section 6.5<br>Page 21<br>Line 661

11. Data availability – authors selected no. However, they have released the database. For full reproducibility, the human labelled subset of the database should be identified or separately released. If this is already the case, it should be mentioned.	Thank you for highlighting this. We have added a separate link for the human-labeled subset in the repository to enhance reproducibility. The full repository, including the human-labeled subset, will be made publicly available once the paper is published.	Section 10.4 and 10.5<br><br>Page 23-24<br>Line : 746-757

**Reviewer # 2**		
**Reviewer's Comment to be addressed**	**Response/Action Taken**	**Document Location**
1. The authors claim limited access in Data Availability, but as far as I know, building the dataset was the main work of this paper. And this dataset was collected from public data, it is the process and methodology of building it that is innovative and needs to be described further in the paper.	Thank you for your thoughtful feedback.<br><br>As noted, the dataset was collected from publicly available sources; however, it was originally unstructured and required extensive processing. Our work focused on transforming this raw data into a structured, annotated dataset, which adds substantial value and uniqueness through our methodological approach.<br><br>To provide clarity on this process, we have included detailed explanations in Sections 3, 3.1, 3.2, and 3.3, with a comparative analysis of our dataset in Section 3.4. Additionally, Figure 1 presents a workflow overview, illustrating the complete process undertaken to create the dataset.	Section 3<br>Page 6-10
2. I think it is a jump to go from a traditional machine learning model and extend it directly to a pre-trained BERT model. The authors could have considered including experiments with CNN, GNN[1], RNN[2], and other models. Or explain the reasons why these methods were not chosen. These methods perform well in both DNA and protein tasks.<br><br>[1]Cao B, Wang B, Zhang Q. GCNSA: DNA storage encoding with a graph convolutional network and self-attention[J]. Iscience,	Thank you for your insightful comment and suggestion.<br><br>BERT-like models were chosen over CNN, RNN [17], and GNN [16] architectures for culinary text classification due to their ability to capture deeper contextual relationships in text through self-attention mechanisms. Unlike CNNs and RNNs, which process data sequentially or in fixed-sized windows, BERT variants analyze entire sentences in parallel and consider both left and right contexts simultaneously. This bidirectional approach allows BERT-like models to understand nuances and dependencies between words more effectively, which is especially valuable in recipe texts where specific ingredient interactions, cooking techniques, and contextual meanings are crucial for accurate classification. Additionally, BERT's pre-training on vast amounts of text data makes it well-suited for	Section 2.2<br>Page 6<br>Line 191-196<br><br><br><br><br>Section 5.1<br>Page 12<br>Line 387-405

2023, 26(3).<br>[2]Li X, Han P, Chen W, et al. MARPPI: boosting prediction of protein–protein interactions with multi-scale architecture residual network[J]. Briefings in Bioinformatics, 2023, 24(1): bbac524.	domain-specific tasks like recipe genre classification.<br><br>While CNNs, RNNs, and GNNs are highly effective in structured or sequential data tasks, such as DNA and protein sequence analysis, they may face challenges in capturing the long-range dependencies and nuanced relationships characteristic of natural language. Recipe texts require models to capture both local and global information, given the variety of ingredient combinations and cooking methods they contain. BERT's self-attention mechanism provides a comprehensive view of these relationships, making it more suitable for this domain compared to traditional models like CNN, RNN, or GNN.<br><br>To address this rationale more fully, references [16],[17] are provided in the last paragraph of Section 2.2, and an in-depth explanation of our choice of BERT-like models is included in Subsection 5.1.	
