# Supplementary material for: Towards automated recipe genre classification using semi-supervised learning
Source: PLoS One. 2025 Jan 28;20(1):e0317697. doi: 10.1371/journal.pone.0317697 (PMC11774350; doi:10.1371/journal.pone.0317697)
Supplement: Attachment — Submitted filename: Response To Reviewers PONE-D-24-34319 v2.pdf [file pone.0317697.s002.pdf]

Response to reviewers' comments of the Journal paper, PONE-D-24-34319		
**Academic Editor**		
**Editor's Comment to be addressed**	**Response/Action Taken**	**Manuscript reference**
**Journal Requirements:**<br><br>Please review your reference list to ensure that it is complete and correct. If you have cited papers that have been retracted, please include the rationale for doing so in the manuscript text, or remove these references and replace them with relevant current references. Any changes to the reference list should be mentioned in the rebuttal letter that accompanies your revised manuscript. If you need to cite a retracted article, indicate the article's retracted status in the References list and also include a citation and full reference for the retraction notice.	We sincerely thank the Academic Editor for their insightful observation regarding the reference list. Following the advice of Reviewer 2 in the previous round of revisions, we added two citations and updated the manuscript accordingly. Additionally, we included one more reference, as detailed in our response to the reviewers' comments.<br><br>The added references, as per the advice of Reviewer 2, are provided below:<br><br>i. Cao B, Wang B, Zhang Q. GCNSA: DNA storage encoding with a graph convolutional network and self-attention. Iscience. 2023;26(3).<br>ii. Li X, Han P, Chen W, Gao C, Wang S, Song T, et al. MARPPI: boosting prediction of protein–protein interactions with multi-scale architecture residual network. Briefings in Bioinformatics.2023;24(1):bbac524.<br>iii. Settles B, Craven M. An analysis of active learning strategies for sequence labelling tasks. In: proceedings of the 2008 conference on empirical methods in natural language processing; 2008. p. 1070–1079.<br><br>Unfortunately, in the previously submitted file titled "Revised Manuscript with Track Changes," the updates in the References section, specifically references 16, 17, and 18, were not highlighted due to an oversight. We deeply regret this oversight and have now ensured that these changes are appropriately highlighted in the "revised manuscript with track changes" for clarity.<br><br>We confirm that no further corrections are required to the reference list beyond addressing this minor error. We sincerely apologize for this inadvertent mistake and hope for your kind understanding and acceptance.	Section Reference Page 25

**Additional Editor Comments:**<br><br>Please double-check to ensure that ALL datasets and codes used in this work can be freely accessed by other researchers for reproduction upon acceptance.	Thank you for your valuable comment.<br><br>We have thoroughly verified that all datasets and codes used in this work are publicly accessible and available in the public domain. The links to these resources have been carefully checked and included in the manuscript, ensuring that they are functional and allow for full reproduction of the work. We are confident that this meets the journal's requirements for reproducibility and transparency.<br><br>3A2M Dataset Link : 3A2M Cooking Recipe Dataset<br>3A2M+ Dataset Link : 3A2M+ Cooking Recipe Dataset<br>Human Annotated Dataset Link : 3A2M Human Annotated Subset<br>Codes Repository Link : Extended 3A2M Project	
